# Selections

**Published:** 1816-04

**Authors:** 


### PART III.

### SELECTIONS.

### FRENCH DICTIONARY OF MEDICAL SCIENCES.

( Continued from p. 271. )

*Article, docimasie pulmonaire.* In our last number, we considered one of the five objections to the *hydrostatic trial of the lungs.* We now proceed to the remaining four.

*Second objection. The lungs may swim, and yet the infant not have respired.* The causes of this phænomenon are *putrefaction, inflation,* or a *particular emphysematous state* of the lungs. — Can *putrefaction,* by causing the developement of gaseous substances, so far augment the specific lightness of lungs, which have not respired, as to make them swim? Upon this question much difference of opinion has existed. Some inaccuracy seems to have arisen from observations having been made upon lungs submitted to putrefaction in a state of isolation from the body; for this forms a condition widely differing from those under which the putrid decomposition of the organ, while inclosed in the chest, is accomplished. Hence, a more conclusive mode has been employed by some experimentalists. Camper, in order to ascertain the point to which putrefaction may proceed in children without causing the lungs to swim, instituted many experiments; and he discovered that, in still-born subjects, the head and extremities might be consumed by putrid decomposition, so far as to render the bones separable by the slightest force, and yet the lungs, just beginning to putrify, not swim. The same result was obtained when the body of the fœtus had been macerated three or four months. The experiments of Schmitt, who may be regarded as the first authority in the history of pulmonary trial, confirm this fact. Yet must we not conclude, from the infrequency of the phænomenon, that it never takes place. Indeed, its occasional occurrence has been incontestibly proved ;* and we are constrained to acknowledge that, "in some rare cases, and under certain circumstances hitherto unknown, lungs, which have never respired, may swim from the mere effect of putrefaction." Are there any means of distinguishing this effect ?

It may be first remarked, that unless putrefaction have proceeded very far, it cannot attack the lungs, of all the viscera least putrescible; *in general*, however, we must add, for Baumer has known an instance wherein the lungs swam from this cause, while universal putrefaction was little advanced. Now, whenever very high putrescency prevails, pulmonary trial must share the uncertainty of all other medico-legal operations in such cases ; and the decision of the court must then rest on other circumstances than those which the body of the deceased presents.

We have only, therefore, to speak of cases where putrefaction is not sufficiently advanced to exclude anatomical inspection ; and here the putrid decomposition commonly confines itself to the surface of the lungs, where are frequently seen striæ formed by bubbles of air, which become disengaged on incision. This is a peculiar, but not constant character of putrefaction ; other more certain signs invariably exist, by which we may determine whether the swimming of the lungs be attributable to putrefaction.

There are some viscera, the specific lightness of which is augmented by putrefaction nearly in the same ratio as that of the lungs which have never respired. Such are, according to Urisberg, the thymus gland, intestines, bladder, and all those organs which in laxity of cellular structure resemble the lungs. Now, as all these are susceptible of swimming from the influence of putrefaction, it is only requisite to submit them also to the hydrostatic trial, in order to determine, from the parity of result between the phænomena exhibited by them and the lungs, whether the swimming of the latter be the consequence of putridity. Another sign, not implicitly to be trusted, because not always appreciable, is the crepitation which arises beneath the scalpel on dividing the surface of the lungs wherein respiration has been accomplished. Putrefaction, even when causing the lungs to swim, does not destroy this sound; and it is never heard in operating on pulmonary organs which have not respired. But the most certain mode of distinguishing the effects of putrefaction from those of respiration, consists in the possibility of expressing with the fingersthe gaseous products of the putrid fermentation. The slices of lung submitted to this process, which have before swum, will

---

* See Kritische Jahrbuecher, &c. Von C, Knape, und A, F. Hecker. Berlin, 1806, Vol. I, Part II, Page 307

afterwards sink if they belong to a fœtus that has not respired; but, in the opposite case, they will continue to swim. The other viscera, rendered lighter than water by putrefaction, will equally sink after expression: hence, the institution of a counter trial should never be neglected. "We see then that the swimming of the lungs, determined by putrefaction, does not at all invalidate the pulmonary trial, when none of the diagnostic means, just pointed out, are neglected."

*Artificial inflation* gives rise to much greater difficulty of distinguishing the lungs, which have respired, from those which have not. Who would not shrink with horror at the idea of exposing the guiltless to infamy and death! How would the juridical physician shudder, if, on announcing that he had discovered upon a fœtus the traces of vitality after birth, the suspected murderer should reproach him with mistaking the effects of maternal tenderness for those of a foul crime, and thus making her efforts to resuscitate her infant, the ground of a dreadful accusation against her. What a situation for an innocent mother!

Roederer denied the possibility of inflating the lungs so as to make them swim. This extraordinary opinion, though contrary to that of many celebrated men, was adopted by some juridical physicians, rather from motives of indolence than conviction. And this error was only dissipated by the authority and experiments of Camper.

Others, without disputing the fact in question, have considered groundless the fear of error to which it may lead. They think that a woman delivered secretly, could not herself execute inflation; and that the circumstance of having been assisted by another would readily be discovered. Again, they deem it improbable that any one, who intends to destroy her infant, should try to resuscitate it. That the first of these suppositions is erroneous, Buttner and Chaussier have conclusively shewn. The second is better founded: for when upon the body of a new born child, mortal injuries evidently inflicted during life, are discovered, and all other circumstances concur to announce the existence of respiration and the crime of infanticide, one can hardly suppose the murderer capable of executing a process to re-animate her progeny. Morgagni admits that pulmonary inflation might be performed by an individual upon the still-born infant of another, with the view of criminating, and destroying, the guiltless parent.

Juridical physicians although agreed that lungs, which have never respired, may swim from inflation, differ as to the manner of appreciating this fact relatively to the hydro-static trial: hence the value of the latter must necessarily be lowered in the eyes of impartial men; and there remained only the hope of detecting in the peculiar characters of inflation and respiration, the means of distinguishing them. As the basis of this inquiry, it has been assumed, that respiration is a vital function, in which not only the lungs, but the osseous and muscular parietes of the thorax participate; that the share which these organs take in respiration is merely passive in inflation; and finally, that, the circulation being modified by the respiratory act, permanent peculiarities after death must necessarily result from these differences of impression; which peculiarities are foreign to inflation. Hence have been regarded, as the especial characters of inflation, 1st. Incomplete dilatation of the lungs; 2ndly. Defect of arching of the thorax; 3rdly. Absence of crepitation on cutting the lungs; lastly, and principally, Emptiness of the pulmonary vessels without previous hæmorrhage. The importance of the subject demands that we examine these data separately.

From the experiments of Schmitt, too numerous to be recorded here, we sum up the following results: 1st. Inflation of the lungs of children still-born, or born in a state of asphyxia, is practicable; 2ndly. It completely succeeds, when duly executed, and no mechanical obstacle opposes the introduction of air; 3rdly. The operation will prove difficult or imperfect, when the respiratory passages are choked with mucus, or the experiment is improperly performed; 4thly. The expansion, spongy state, and rose-colour of inflated lungs, and their faculty of swimming, will vary according to the degree of success of the operation, and these properties manifest themselves in the direct ratio of the quantity of air which has penetrated; 5thly. Crepitation takes place on incising inflated lungs, if the operation have at all succeeded, and on compressing the lungs between the fingers, a white bloody froth is seen to escape from the places of incision; 6thly. Inflation always determines an elevation of the chest and abdomen, and the dilatation of the former may be appreciated after death; 7thly. Inflation, the most perfect, cannot sensibly augment the weight of lungs which have not respired; and, in most cases, the relation between the weight of inflated lungs and that of the body, is the same as in infants which have not respired.

In opposition to these opinions, it has been thought that the lungs cannot be completely distended by inflation; and that, from the structure of the bronchiæ which opposes a greater obstacle to the introduction of air by the left lung than by the right, it would be impracticable fully to inflate the left lung. Schmitt acknowledges the general difficulty of the operation, but proves by experiment, that it is not always impracticable: and Chaussier has observed, in children who have respired some hours, the left lung completely dilated, while the right was almost, or entirely, destitute of air.* Secondly, According to Metzger, inflated lungs are not crepitous; and herein he thinks, that the effects of inflation may be distinguished from those of respiration; but this opinion is unsupported by any physical reasoning, and is contradicted by numerous facts. Thirdly, Metzger has also established, as a character of inflation, the absence of arching of the chest, but erroneously; since this forms one of its most constant signs: and, even if we admit, with Loder, the impossibility of inflating lungs

* Considérations Médico-légales sur l'Infanticide.

which have not respired so as to determine a dilatation of the thorax equal to that produced by the respiratory act, no other consequence can be drawn from it than *that dilatation of the chest caused by inflation, and dilatation determined by the respiratory process, offer only a relative difference.* Now, it must always be very difficult to appreciate the degrees of this difference: and it has been observed by Schmitt, that there may exist a very slight dilatation of the thorax in infants who have respired some time, but imperfectly; that, in some cases, the lungs are completely distended, and yet the thorax not dilated; and again, that the dilatation of the thorax may be very apparent when the lungs contain little or no air.

The sign, by which the effects of inflation may be most certainly distinguished from those of respiration, is the emptiness of the pulmonary vessels, provided it has not arisen from hæmorrhage. Yet, although the physiological doctrine upon which this phænomenon is explained, be incontrovertible, many difficulties here present themselves. Ocular inspection, like most of the proposed methods of ascertaining the quantity of blood that issues from the lungs, and the diameter and distension of the great pulmonary vessels, must be exceedingly fallacious. Indeed, Plocquet's trial by weight constitues the only mean of determining rigorously the quantity of blood which has penetrated into the lungs, and deciding the question whether respiration or inflation have distended those organs. We shall revert to it presently.

In order to shew how far inflation may produce phænomena resembling those left after death, by the respiratory act, we quote the following cases from a foreign Journal.

Case I.—On February 9, 1812, a woman was delivered, by the forceps, of a male infant at maturity, weighing eight pounds two ounces; and twenty inches six lines in length. Pulsations of the umbilical arteries and heart were the only sign of life exhibited. They continued more than half an hour; and, during this time, air was repeatedly inflated into the lungs, with care to imitate respiration by compressing the chest; but this experiment, as well as every other, was fruitless: once only, affusion (of water) caused contraction of the abdominal muscles without the slightest dilatation of the chest. *Upon dissection*, a few days after, the body presented no trace of putrefaction; the chest was vaulted externally; on opening it, the lungs were found distended, the inferior left lobe stretched towards the anterior part of the thoracic cavity; the colour of the lungs bordered on clear red, their surface was marbled; the diaphragm was very little arched; the lungs with the heart and thymus gland, swam in a vessel of fresh water; blackish blood was found in the ventricles of the heart; the lungs, separated from that organ and the thymus, weighed one ounce, six drachms, and two scruples; consequently, the weight of the lungs was to that of the whole body, as 1 to $76\frac{1}{11}$; placed alone on water, they swam, as did each lung separately; they contained, on division, little blood, and were distinctly crepitous; bubbles of air escaped in abundance on dividing them under water; all the fragments without exception, swam, even after having been strongly compressed with the fingers; which operation caused the oozing of black blood.*

CASE 2.—The infant here was a male, still-born, at the full time; six pounds fourteen ounces six drachms in weight, twenty inches long. No sign of life was perceptible. Appearances indicated that it had been some days dead. The lungs were inflated by a tube passed through the nostril. The phænomena, both external and internal, displayed on anatomical inspection, varied so little from those noted in the first case, that we need not particularize them. The relative weight of the lungs to that of the body, was as 1 to $67\frac{2}{7}\frac{1}{0}$.

Lastly, the lungs may display *a peculiar emphysematous state*, dependent neither on putrefaction nor inflation. It is observed, according to Chaussier, in infants which exhale no putrid smell, and whose organs preserve a natural colour and consistence. He, several times, has found that a part of the lungs of children, extracted by the feet, swam, especially where the mother's pelvis was strait, although respiration had certainly never taken place: and he is disposed to attribute the phænomenon to some contusion inflicted on the lungs during extraction, whereby blood is effused into their structure; from the decomposition of which bubbles of air are extricated. This explanation appears to acquire probability from the brownish violet hue which the lungs, under such circumstances, exhibit. However it be, this accidental lightness of the lungs may be readily distinguished from the aëriform fluid being contained in their laminous structure, whence it will issue on compression; and, this done, the lungs will directly sink in water; which would not happen, if the air were contained in the bronchial cells.

*Third Objection. Demonstration by the hydrostatic trial of the lungs that a fœtus has not respired, affords no proof that it has not lived.* This objection, taken in a certain sense, is real. Many functions, connected with organic life, may be prolonged, for a time, with a new-born child, whom various causes have prevented from respiring. Among these causes are, constitutional weakness; obstruction of the air-passages; malformation of the chest or abdomen; the fall of the fœtus into a liquid immediately on expulsion; or privation of air, in consequence of the membranes remaining entire. This result may the more readily be produced, since Buffon and others have established, on well-known physiological principles, the conclusion, that causes, capable of exciting prompt asphyxia in animals which have enjoyed, during a certain time, extra-uterine life, are not always adequate to its produc-

* Journal der Practischen Heilkunde, von Hufeland, August, 1812.

tion in the new-born. Moreover, all these obstacles will readily be distinguished on the body; and, in doubtful cases, we must always lean to the side of mercy. Under these circumstances, the innocent can never be endangered by the pulmonary trial. Before we terminate this subject, it may be observed, that obstruction of the air-passages by mucus or liquor amnii, frequently prevents the due establishment of respiration in new-born children, and thus induces death. It little matters how the liquor amnii gets introduced: the fact is unquestionable and common: and hence the juridical physician should never lose sight of it in examining a fœtal subject, but distinguish the obstruction by mucus or liquor amnii from that consequent on the introduction of foreign liquids; especially when the question regards a fœtus found in a privy or other such situation. Schéel has established the following rules on this subject: When the liquid contained in the trachea is limpid, contains no bubbles of air, or is not converted into froth, we may certainly conclude that the infant has not respired. If, on the contrary, it consists of froth, an inference may be drawn that respiration or inflation has taken place. Lastly, when this liquid contains much mucus or meconium, or is thick and tenacious, the fœtus, although born alive and able to respire, must necessarily perish, from the imperfection of that process. Schmitt, while he adopts these precepts, thinks that the presence of air-bubbles in the liquid contents of the trachea and bronchiæ does not conclusively indicate respiration or inflation, unless the phænomenon concur with others characterizing these processes. Indeed, we may readily conceive that certain morbid conditions will cause the developement of gaseous substances in the products of pulmonary exhalation, independently of the introduction of external air.

*Fourth Objection. A new-born child may have respired, and yet the lungs not swim.* Several facts of this kind have been recorded by different authors.* In a case, mentioned by Heister, the child lived nine hours, moved, swallowed liquids, and cried feebly. The inconsistency of this phænomenon with the best established laws of physiology rendered such observations suspicious; and they were nearly forgotten, when the celebrated Loder found in a fœtus of seven months, which had lived two months after birth, and repeatedly exerted its vocal organs, the lungs very compact, of a brown red colour, and in all respects like those that have never respired. Submitted, whole and piece-meal, to the hydrostatic trial, they did not swim. No morbid condition was observed to account for this circumstance. Other physicians, equally creditable, have since reported similar cases. In examining these observations, we find, that none of the subjects of them were at the full term, and that *absolute* submersion of the

* Zeller, Mauchart, Heister, Torrez.

lungs (submersion of the organs whole and piece-meal) only took place in children which presented *all* the characters of immaturity; and again, that with those in whom, from having been born after the seventh month, these characters were less decided, some fragments of the lungs always swam. Facts so extraordinary may be thus explained: Life is occasionally prolonged for a time with the fœtus, although respiration be very imperfectly performed; and this possibility is greater in proportion as the period of maturity is more remote. Again, in some rare cases, respiration may be so feeble, that the air penetrates only into the trachea, or first ramifications of the bronchiæ. Such was the opinion of Haller. Now this imperfect respiration does not preclude the possibility, that the small volume of air which has reached the trachea, may, in traversing the glottis upon expulsion, produce the sounds frequently heard in these cases. What has been before said respecting the tardiness with which asphyxia takes place in new-born animals, should also be recollected; and hence we may conclude, that *tracheal respiration* will suffice to support life, for a time, in an infant recently expelled; but the period must soon arrive when this imperfect process will no longer avail, and death inevitably ensue.* This event will happen sooner in proportion as the fœtus is nearer the period of maturity, and the mechanical obstacle to respiration more considerable.

Sanguineous congestion of the lungs, determined by suffocation, is another cause thought to be capable of producing submersion of these organs in the fœtus which has respired. But this phænomenon has never been observed by the most experienced juridical physicians; and all error, in such a case, might be avoided, by expressing from the lungs, cut in pieces, the superabundant quantity of contained blood.

*Fifth Objection. A fœtus may have respired, and yet not lived.* This objection appears paradoxical, and contrary to the principle in forensic medicine, which teaches us to consider respiration as the signal and proof of animal life. Yet, in order to complete the history of pulmonary trial, we shall do well to notice it. Dr. Benedict, of Chemnitz (Medico-Chirurgical Gazette of Salzbourg, Dec. 1812), asserts, that in an hydro-cephalic fœtus, born at the full period, with decided malformation of the head and brain, the lungs exhibited all the phænomena of perfect respiration. But this fact rests upon suspicious evidence; since the mother was delivered without witness, and it was her obvious interest to make the magistrate believe that the infant was still-born. Hence we shall not transcribe Dr. Benedict's explanation. In a still-born hydro-cephalic subject, since examined by Marc, the lungs displayed all the characters which indicate the non existence of

---

* The whole of this hypothesis appears to us extremely questionable.
EDIT.

respiration. But even supposing that, in such a subject, whose malformation implies the impossibility of extra-uterine life, respiration, as admitted by Benedict, may be established, the only consequence is, that to such cases the trial of respiration is inapplicable. The validity of this process cannot be shaken by an exception which never presents itself in a fœtus regularly constructed, and capable of supporting an independent existence.

*Appreciation of Plocquet's trial.* However ingenious this process may at first sight appear; however firm the principle upon which its theory rests; it is less conclusive than the hydrostatic trial, for the stereometric relations of bodies are not constantly the same. This variation of the relations of weight between the lungs and body was first demonstrated by Jaeger. He has proved, that the mere difference of sex serves to make these data vary: hence it is necessary first to determine the maximum and minimum of the relations of weight, absolute and relative, not only between the lungs and body of the male child, but those of the female also. Again, the partial nutritive activity of organs is so irregular as to induce numerous anomalies in these relations; and the various degrees of obesity would alone suffice to destroy the certainty which it is so essential to acquire. To this circumstance may be principally attributed the remarkable difference between the results of Plocquet's researches, and those of the Danish physician, Harttmann. According to the latter, the mean relation between the total weight of the body and lungs of a fœtus which *has* respired, will be as 48,971 to 1; and of a fœtus which *has not* respired, as 59,839 to 1. We may summarily add, that the relations fixed, after multiplied experiments by Chaussier and by Schmitt,* confirm the inferences of Harttmann rather than those of Plocquet: and, what still more conclusively demonstrates the fallibility of Plocquet's process, Chaussier has proved, beyond doubt, the possibility of meeting with the relation of 1 to 70, or more, between the lungs and body of an infant which *has* respired, and the relation of 1 to 35, between those of an infant in whom respiration *has not* taken place. Thus it appears, that the trial by weight is not so certain as to be entitled to the unlimited confidence which some have reposed in it, or to claim superiority over the hydrostatic trial.

The calculations of Schmitt, moreover, shew, that we may regard as the opposite extremes of the absolute weight of the lungs:

---

* From tables given by Marc, which we have not room to copy, it appears that Schmitt has placed the mean relation of the weight of the body to that of the lungs, *in children which had respired*, as $42\frac{528}{1130}$ to 1; Chaussier, as $39\frac{119}{1225}$ to 1:—Schmitt, *in children which had not respired*, as $52\frac{879}{1035}$ to 1; Chaussier, $49\frac{9}{1109}$ to 1. Edit.

| | Grams. | Centigrams.* | |
|---|---|---|---|
| The weight of | 18 | 20 | In infants, prematurely born, which have respired. |
| and of | 78 | 52 | |
| The weight of | 18 | 65 | In infants, prematurely born, which have not respired. |
| and of | 74 | 91 | |
| The weight of | 35 | 65 | In children, born at full time, which have respired. |
| and of | 109 | 6 | |
| The weight of | 35 | 20 | In children, born at full time, which have not respired. |
| and of | 84 | 30 | |

*Application of the preceding details to the practice of forensic medicine.* This section contains nothing very important. The three only serious objections to the hydrostatic trial are, 1st. The possibility that a fœtus may respire before birth; 2dly, That swimming of the lungs may be determined by inflation; and 3dly, That respiration may have been accomplished, although the lungs do not swim. The other objections have been sufficiently combatted.

But even the three principal objections to the hydrostatic trial just stated, and the instability of Plocquet's experiment, are not sufficiently great to proscribe the introduction of these processes into forensic medicine: yet the difficulties attendant on them, demand from the juridical physician especial prudence and circumspection: and we may conclude, that "if in the greater number of cases the existence or non-existence of extra-uterine life cannot be positively pronounced, there are many others in which the question will be more readily decided."

This will happen particularly when the data, obtained from the respiratory trial, concur to prove that respiration has *not* taken place after birth, provided there exist not the only circumstance, capable of leading here into error, viz. the possibility that the lungs may sink although the fœtus has respired. But we should recollect, that this phænomenon has been observed in individuals bearing all the traces of immaturity or marked constitutional weakness: and with such subjects only, not with those whose constitution and physical developement were perfect, could the results of pulmonary trial, which announced the absence of respiration, be at all doubtful. The other causes, capable of producing submersion of the lungs after respiration, as morbid alteration of their substance, obstruction of the air-tubes, and sanguineous congestion, need hardly be traced here: and, in a criminal affair at least, if error be committed, that which occurs on the side of mercy is far least to be dreaded.

"When, indeed, the results of anatomical inspection lead to the presumption that respiration has taken place, the dread of

* Eighteen grains make one gram; five centigrams, one grain; French weight, EDIT.

sacrificing an innocent person should increase our hesitation and reserve. Then, especially, we should not lose sight of the two principal circumstances which might cause the fœtus which has not respired after birth, to be mistaken for one that has respired, viz. *respiration during delivery, and inflation.* Absolute certainty, in this respect, cannot always be acquired; but there are many cases in which it may be obtained from concurrence of the results derived immediately from the state of the organs interested in the respiratory act, with the instructions gleaned on the mode of delivery, and with the other phænomena which the body may present.

"*Definitive Conclusions.* It results from the preceding observations,

"1st. That the process of Daniel is too complex, and requires attention too minute, and instruments too exact, to be introduced into medico-legal practice.

"2dly. That of all the pulmonary trials, the hydrostatic is to be preferred.

"3dly. That neither it, nor any other, is capable of determining the slightest degree of respiration; and that, in such cases, it is only applicable, as affording exculpatory evidence.

"4thly. That, in order to prove respiration, and consequently life, after birth, the pulmonary proof should coincide with the following circumstances:—A. The fœtus should present all the signs of maturity.—B. It ought not to be attacked by putrefaction, so far as to preclude research, or render it uncertain.—C. It should exhibit no malformation to which death may be attributed.—D. The head should present, neither externally nor internally, any condition which may have determined death during the birth.—E. The assemblage of signs, indicated in the course of this article, and drawn from the state of the lungs, thorax, diaphragm, and abdominal viscera, should exist so as to prove that respiration has been complete.—F. The legal documents (in a case of child-murder) ought to substantiate that there has been no inflation.—G. The information as to what passed during delivery should exclude all supposition that the infant had respired before birth.—H. Traces should exist upon the fœtus, indicating that it has been the victim of criminal procedures.

"5thly. That Plocquet's trial by weight can serve only to complete the inferences drawn from the hydrostatic process, inasmuch as the results, obtained from the execution of both, correspond. Notwithstanding the inconstancy of the results afforded by Plocquet's trial, they are, when the experiment is made upon a full-grown fœtus, in most instances, nearly such as the inventor of the process has established. We need not indicate the conditions under which the pulmonary trial may prove that the fœtus has not respired. They necessarily result from the preceding article."

The principal monographs on pulmonary trial are those of *Zeller*, 4to. Tubingen, 1691; *Heister*, 4to. Helmstadt, 1722; *Zeller*, 4to. Halle, 1725; *Geelhausen*, 4to. Prague, 1728; *Al-*

*berti*, 4to. Halle, 1728; *Goelicke*, 4to. Frankfort, 1730; *Heister*, 4to. Helmstadt, 1732; *Schoepfer*, 4to. Rostock, 1733; *Kaltschmied*, 4to. Jena, 1751; *Schmiedel*, 4to. Erlangen, 1763; *Koenig*, 4to. Halle, 1772; *Daniel*, 4to. Halle, 1780 (with an Appendix added by *Schultz* in 1787, and since enriched by the Dissertations of *Meckel* and others); *Loder*, 4to. Jena, 1779; *Demanche*, 4to. Rheims, 1779; *Jaeger*, 4to. Ulm, 1780; *Plocquet*, 8vo. Tubingen, 1782; *Mayer*, 4to. Frankfort, 1782; *Chaussier*. 4to. Dijon, 1786; *Scholl*, 4to. Stutgard, 1786; *Metzer*, 4to. Konigsberg, 1783 — and another, 4to. Konigsberg, 1787; *Kieffer*, 4to. Jena, 1788; *Aasheim*, 4to. Copenhagen, 1791; *Olberg*, 4to. Halle, 1791; *Meckel*, 4to. Halle, 1802; *Olivaud*, 8vo. Paris, 1801; *Homann*, 4to. Helmstadt, 1807; *Chaussier*, 8vo. Paris, 1808; *Lecieux*, 4to. Paris, 1811; *Devolder*, 4to. Paris, 1812.

On this subject may also be consulted with advantage, a dissertation on a work, entitled, *Nouveau Recueil de differens Traités de Médecine*, Paris, 1744; a memoir by *Portal*, *Memoires de l'Academie Royale des Sciences*, for the year 1769; *Frank*, *System einer Medicinischen Polizey*, 1794; *Metzger*, *System der gerichtlichen arzneywissenschaft*, 1798; *Mahon*, *Médecine légale et police Médicale*, 1811; *Foderé*, *Traité de Médecine légale et d'Hygiène publique* (a new edition of this work has recently been published); the article, *Medicina Forensis*, in *Dr. Parr's* Dictionary; and an *Essay*, from the French of *Mahon*, by *Johnson*. Dr. Male, of Birmingham, has also recently published an *Epitome of Juridical or Forensic Medicine*, which we shall take an early opportunity of reviewing. EDITORS.

### "*Case of Aneurism of the Cœliac Trunk, with remarkable Disproportion in the two Ventricles of the Heart.*"*

"A PASTRY-COOK, aged thirty-three, of sanguine temperament and strong constitution, was, in the year 1800, admitted into *L'Hotel Dieu*, on account of illness. Tightness of respiration and hæmoptysis, were the only symptoms of which he had any recollection. The affection lasted three or four months, and he was bled twenty times during that period. He afterwards enjoyed good health, except that he was subject to nasal hæmorrhage: but respiration remained free, and he never experienced a sense of suffocation either in walking quick or climbing stairs."

"In June, 1804, he was attacked with universal pains, which he compared to the pricking of pins; and felt pulsations in the epigastric region which gradually increased. After some time, his digestion became deranged; and thenceforward, even a little food would induce a sense of weight at the stomach, and inclination to vomit. He was frequently obliged to desist from eating; and, the uneasiness ceasing in about fifteen minutes, he generally resumed his meal, but the food was sometimes rejected by vomiting."

* Communicated by Dr. Nysten.—*Journal de Médecine, par Leroux.*

"To the pungent pains and epigastric pulsations, were added colics, becoming progressively more violent. The patient lost flesh, but continued to work till January 13, 1805, the day of his admission into *L'Hopital de la Charité.* To this measure he was incited rather by the colic and indigestion, than by the pulsation in the epigastric region. The colic was soothed by rest and regimen; the appetite returned; and the pricking pains were confined to the flanks and loins. On the 13th of February he presented the following symptoms:"

"Little emaciation; countenance somewhat florid; tongue clean; appetite good; no thirst. Pulsations in the epigastrium strong, isochronous with those of the pulse, perceptible to the extent of two or three inches, and alternating with the stroke of the heart, which was well marked, but disappeared when the patient lay on his right side. Respiration natural in every change of posture; reclination on the sides, easy; on the abdomen impracticable; pricking pains, at times, in the flanks and loins, particularly in the supine posture. Abdomen, not painful on pressure; no tumor in the epigastrium; pulsations of this region aggravated at different hours during the day, and often in the night. No fever; little sleep; pulse regular, full, somewhat vibrating."

"February 15th. Same state. On the following days the epigastric pulsations were stronger:

21st. They were perceptible to the eye, and a round projecting tumor, two or three inches in extent, was detected in the epigastrium. This tumor was strongly marked when the patient lay on his right side, but he could not remain long either in this posture or upon his back; hence he generally reclined on the left side. Pains were felt in the parietes of the chest, resembling the stabbings with a knife. Respiration free; countenance less florid than before, neither injected, nor wearing the expression commonly observed in active aneurism of the heart. Tongue clean; good appetite; no thirst or fever. Pulse regular, vibrating, completely synchronous with the epigastric pulsation, and alternating with the stroke of the heart."

"February 22. Epigastric tumor painful on light pressure. Lumbar pains; appetite good."

"Feb. 23. Respiration now frequent. The functions of the diaphragm seemed to be impeded by the increase of the tumor, the pulsations of which were become tumultuous. The pains of the flanks and loins continued. The vibration of the pulse was such that the strokes of the radial, brachial, carotid, external maxillary, and temporal arteries were distinctly visible; all of which were synchronous with those of the tumor, but alternated with the stroke of the heart. Night sleepless, and lancinating pains felt in the flanks, loins, and chest."

"Feb. 24th. Symptoms continued. No cough or expectoration. Appetite unimpaired; tongue clean; evacuations natural; night sleepless."

"Feb. 25th, morning. Same state. Death took place suddenly, and without a struggle, at eleven o'clock at night."

"DISSECTION."

"*External appearances.* Face and lips much discoloured. No tumor perceptible to the sight or touch, in the epigastric region. Little emaciation; no serous effusion."

"*Cranial Cavity. Dura Mater* little injected; some serum in the cavity of the external arachnoid membrane.* Cerebral substance soft; medullary, sprinkled with small red puncta. Half a drachm of serum in each lateral ventricle."

"*Thoracic Cavity.* Pleura sound: lungs unadhering, crepitous, marbled on the surface, pink internally. Pericardium containing two drachms of transparent serum. Heart of the ordinary volume, but the capacity of the aortic ventricle as large again as that of the pulmonary: the parietes of the former exhibiting a very firm structure, a thickness of nine or ten lines about the middle of the organ, and of six or seven towards its base and apex. All the cavities contained black liquid blood; the right, in the greatest quantity. A fluid of the same kind was found in the vena cava and aorta. The latter vessel presented at its arch two small dilatations which formed little eminences externally.† Otherwise, the thoracic aorta was sound."

"*Abdominal Cavity.* About two pounds of black blood, almost wholly coagulated, covered the transverse colon, stomach, and duodenum: some of it had also penetrated to the posterior part of the hypochondria. The omentum was tinged by it so as to appear inflamed. The blood, covering it, was traversed by filaments which might have been mistaken for the membranous bands produced by inflammation, but evidently formed by the fibrine of the blood, and readily separable from the parts to which they seemed adherent."

"After having washed away the blood, we discovered, in the midst of the epigastrium, a circular tumor from four to five inches in diameter, situated between the liver, stomach, and duodenum. This tumor had thrust the stomach towards the left hypochondrium, and pushed anteriorly its pyloric extremity, and the first curvature of the duodenum. It was, moreover, covered by a thin membrane, apparently belonging to the peritonæum; below which was found a layer of sero-albuminous matter, whitish, and demi-solid: the pancreas, without being perceptibly altered, adhered to the subjacent parts of the tumor. Upon its superior and anterior part, a circular opening was seen, with a thin, fringed, and blackish margin, into which the extremity of the little finger might be introduced. From this orifice, the blood had been effused

* Bichat called all that portion of the arachnoid between the dura and pia mater, the *external arachnoid;* and asserted that, like the other serous membranes, it forms a sac without an opening.—EDITORS.

† Evidently the commencement of aneurism.—EDITORS.

into the abdomen. It communicated with a cavity formed in the interior of the tumor. This, having been examined, was found to arise, by a broadish base, from the abdominal aorta, above the superior mesenteric artery, and to be formed by an aneurismal dilatation of the cœliac trunk. On dividing it vertically to the interior of the aorta, the latter was seen to be quite sound. The capacity of the aneurismal sac might be about two inches in every direction. It presented no marked contraction at the place of its communication with the aorta; so that the cœliac trunk was dilated from its origin. It was filled with coagulated blood, more solid in consistence as it approached the parietes of the sac; where there were fibrinous concretions, of a yellowish white colour, deposited in lamellated strata; the exterior of which were the most solid and destitute of colour, and adhered so strongly to the internal surface of the sac, that they could with difficulty be separated. Upon clearing the parietes of the aneurism, they were seen to be composed of the internal membrane of the artery, of its fibrous coat, and of a condensed external cellular texture, and to be not more than a line in thickness. The hepatic, splenic, and coronary gastric arteries arose from the sac, but exhibited no dilatation. The abdominal aorta contained a little black fluid blood. No depression was observed in the vertebral column at the part corresponding to the tumor. The stomach was small but sound. The intestines, spleen, liver, pancreas, kidnies, and bladder, were in the natural state."

The preceding case appears to us uncommonly interesting. Cœliac aneurism is of rare occurrence: and, as no doubt can be entertained of the correctness of M. Nysten's observation, we have here an additional evidence of the inapplicability of Scarpa's doctrine respecting the formation of aneurismal sacs, to *every* case of aneurism.—Editors.

